# Infectious abortions in small domestic ruminants in the Iberian Peninsula: Optimization of sampling procedures for molecular diagnostics

**DOI:** 10.3389/fvets.2023.1152289

**Published:** 2023-03-09

**Authors:** Oihane Alzuguren, Lara Domínguez, Gema Chacón, Alfredo A. Benito, Oscar Mencía-Ares

**Affiliations:** EXOPOL S.L., Zaragoza, Spain

**Keywords:** abortifacient pathogen, *Chlamydia abortus*, *Coxiella burnetii*, fetal tongue, goat, placental swab, qPCR, sheep

## Abstract

**Introduction:**

Infectious abortions have a major impact on small domestic ruminant farms, i.e., sheep and goats, both in terms of profitability and health status. Therefore, rapid and sensitive diagnosis is essential to minimize losses. Currently, molecular techniques, such as qPCR, are routinely used for their diagnosis, which imply the need to manipulate all abortive material, with consequent biosafety risks. Here, we evaluate the frequency of the main abortifacient pathogens in small domestic ruminants in the Iberian Peninsula and also assess an alternative approach for the optimization of sampling for molecular diagnosis.

**Results:**

A total of 392 clinical cases were analyzed from April 2020 to May 2021, evidencing that the main causative agents of abortion detected were *Coxiella burnetii* (49.0%), *Chlamydia abortus* (38.3%) and, to a lesser extent, *Toxoplasma gondii* (10.2%), *Salmonella enterica* (7.1%) and *Campylobacter* spp. (6.1%). An uneven distribution of these pathogens was observed between ruminant species, with a higher frequency (*p* < 0.05) of *T. gondii, S. enterica* and *Campylobacter* spp. in sheep than goat abortions, and among geographic areas, highlighting the higher frequency (*p* < 0.05) of *T. gondii* and *Campylobacter* spp. in the north compared to southeastern Spain. The alternative sampling method, consisting on the use of fetal tongues and placental swabs in replacement of the whole fetus and placental tissue, offered a very good agreement with the classical method for all pathogens, except for low concentrations of *C. burnetii*, which seems to have a doubtful role in abortion when its concentration in the abortifacient material is low.

**Conclusions:**

This study reveals a high frequency of infectious etiology in abortions of small domestic ruminants in the Iberian Peninsula and validates for the first time an alternative sampling method for molecular diagnosis that will help to provide rapid and accurate results while minimizing biosafety risks.

## Introduction

Abortions have a strong impact on the health status and profitability of sheep and goats production (1, 2). These clinical processes also have important implications for public health, since most of the abortifacient pathogens are considered potentially zoonotic, with a particular risk for veterinary practitioners, farmers and laboratory staff (3). Indeed, these agents can cause both abortions in women and epidemic outbreaks (4), such as the Q Fever epidemic in The Netherlands in the period 2007–2010, which was associated with abortion storms in goats caused by *Coxiella burnetii* (5).

The main infectious agents causing abortions in small ruminants are *Chlamydia abortus, C. burnetii, Toxoplasma gondii, Salmonella enterica, Campylobacter* spp., *Brucella* spp. and Pestivirus (6–10). The distribution of these abortifacient pathogens may vary among regions and flocks worldwide attending to several factors, such as management practices, flock size, climatic conditions, vaccination, eradication strategies or nutritional factors (11–14).

Detection of the causative agents of abortions in small ruminants should be performed in a systematic investigative approach of fetal organs and placental samples due to the differential tissue tropism of each pathogen (8). This makes it difficult to standardize laboratory techniques for the detection of multiple causative agents, requiring the whole fetus and placenta from the abortion to be sent to the diagnostic laboratory. There, biological samples are manipulated to select the most appropriate tissues for pathogen detection and pools of different tissues are performed to optimize the detection of all causative agents. This not only increases the biosecurity risk, given the zoonotic nature of many of these agents, but also cross-contamination between samples and a reduction in the number of abortions processed in the laboratory. Given these limitations, alternative sampling methods have been developed with partial success, to facilitate the etiologic diagnosis of abortions in small ruminant, such as the fetal oropharyngeal swabbing and puncture of the fetal lung (15).

In this study we aimed to assess the frequency of the main causative agents responsible of abortions in small domestic ruminants in the Iberian Peninsula from 392 clinical cases referred to a veterinary diagnostics laboratory from April 2020 to May 2021, and also evaluate the suitability of fetal tongues and placental swabs as an alternative, rapid and sensitive sampling procedure for the detection of abortifacient pathogens in a selection of 98 of these clinical cases.

## Materials and methods

### Clinical cases of abortions referred for molecular diagnosis

**Figure 1:**
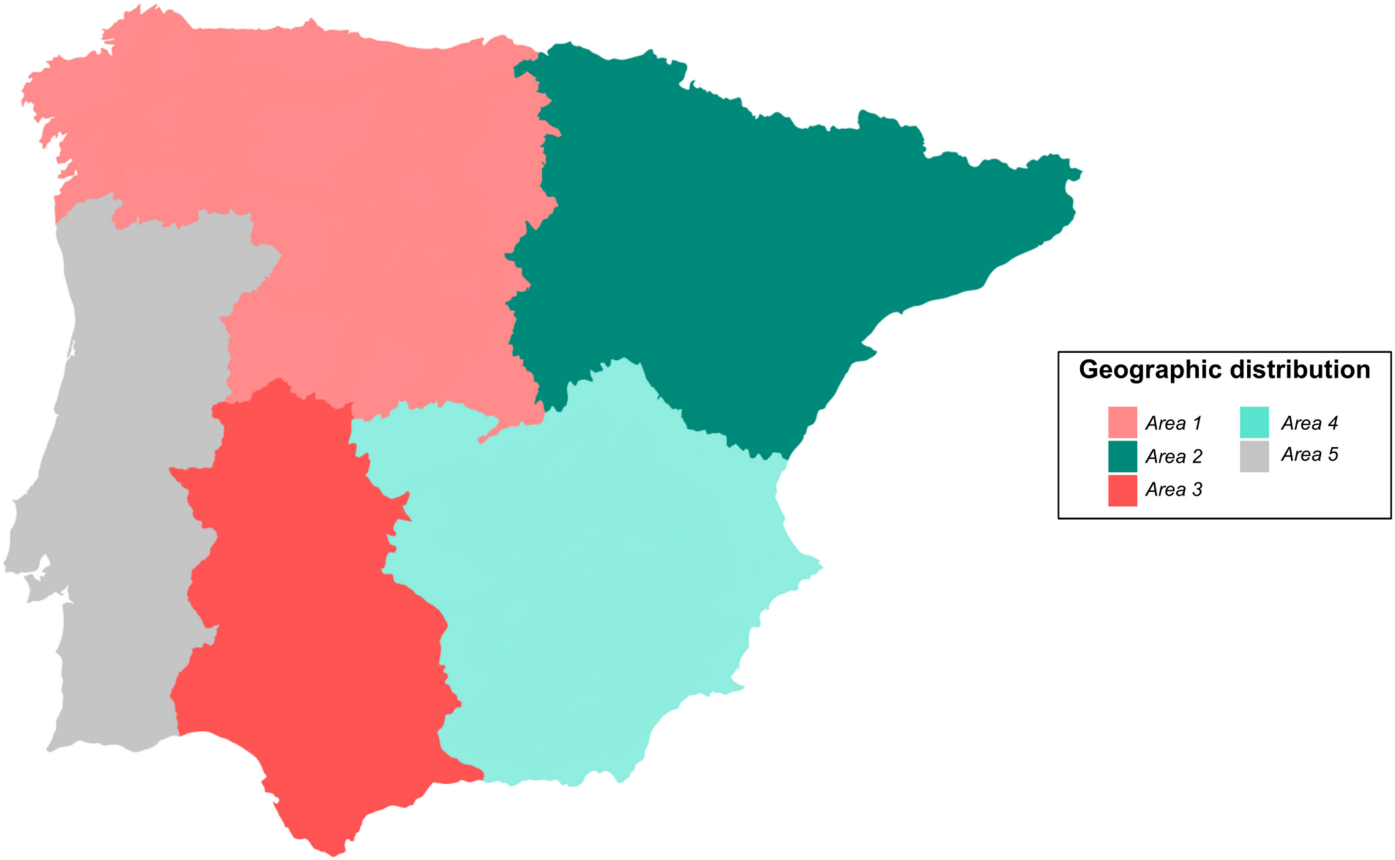
Geographic distribution of the Iberian Peninsula into areas referring 392 clinical cases of abortion in small domestic ruminants to the diagnostics laboratory between April 2020 and May 2021. Area 1: Northwestern Spain; Area 2: Northeastern Spain; Area 3: Southwestern Spain; Area 4: Southeastern Spain; Area 5: Mainland Portugal.

Small domestic ruminant abortions (*n* = 392), i.e., sheep (302) and goats (90), were referred to the veterinary laboratory Exopol S.L. (Zaragoza, Spain) for molecular diagnosis between April 2020 and May 2021. These cases were received from 333 clinically affected farms that had reported abortions. These farms were distributed throughout the Iberian Peninsula, with cases reported from 39 Spanish provinces and 5 Portuguese districts. Based on the geographical location, these regions were divided into five different areas (Figure 1), i.e., northwestern Spain (Area 1; 84 clinical cases), northeastern Spain (Area 2; 88 clinical cases), southwestern Spain (Area 3; 89 clinical cases), southeastern Spain (Area 4; 101 clinical cases) and mainland Portugal (Area 5; 30 clinical cases). Cases from Spanish and Portuguese islands were excluded from this study.

### Sample collection and processing

From each clinical case, samples of placenta, fetus or both, when available, were collected by the veterinary practitioner and referred to the laboratory for further analyses. When more than one abortion was referred from a flock at a time, each placenta and fetus was individualized for diagnostic procedures and the results were further provided at the case level for the frequency study, combining the findings of the individual cases.

Samples of liver, lung, brain, abomasal content and tongue tissue were collected from the fetus using sterile forceps, scissors and Pasteur pipettes. From placenta, placental tissue and a placental swab from the cotyledons and inter-cotyledonary area were collected for further analyses. All samples were collected under aseptic conditions to avoid cross-contaminations. These samples were processed in the laboratory differently depending on whether a classical or alternative approach was used.

#### Classical sampling procedure

The classical sampling procedure consisted of pooling liver, lung, brain, abomasal content and placental tissue using 25 mg of each sample and placing them together in DNAse/RNAse-free microtubes with 1 mm zirconium beads and 400 μl of phosphate-buffered saline (PBS) 1X and subsequently homogenized using the MagNA Lyser automated tissue homogenizer (Roche, Mannheim, Germany) for 2 cycles of 25 s at 6,000 rpm. This procedure was used to assess the frequency of abortifacient pathogens. For the comparative study, placental tissue was individualized from other tissues and processed following this protocol for further comparisons.

#### Alternative sampling procedure

The alternative sampling procedure consisted of individual processing of fetal tongue tissue and placental swabs. Fetal tongues were processed following the protocol described in the previous section for solid tissues. For placental swabs processing, these were placed in microtubes containing 700 μl of PBS 1X and vigorously homogenized to guarantee cell release. These microtubes were centrifuged at 13,000 g for 5 min for pellet recovery. Pellets were further transferred to a microtube containing 200 μl of PBS 1X and homogenized at 6,000 rpm for 25 s in the MagNA Lyser homogenizer.

### DNA/RNA extraction and pathogen identification by quantitative PCR

DNA/RNA extraction was performed following the manufacturer's instructions using the MagMAX™ Pathogen RNA/DNA commercial kit (ThermoFisher Scientific, Massachusetts, USA) and the KingFisher Flex automated magnetic particle processor (ThermoFisher Scientific, Massachusetts, USA).

*T. gondii, C. abortus, C. burnetii, Campylobacter* spp., *S. enterica, N. caninum* and Pestivirus identification and quantification were performed by qPCR/RT-qPCR in pooled fetal tissues, fetal tongues, placental tissues and placental swabs using commercial qPCR kits (EXOone qPCR Kits, Exopol S.L., Spain) and following manufacturer's instructions. These assays target the repeat region B1, *ompA* gene, *IS111* sequence, *gyrB* gene, *ttrC* gene, *NC5* gene and *5'UTR* region, respectively.

The amplification results of the qPCR/RT-qPCR assays were expressed in a semi-quantitative form with the cycle of quantification (Cq). The threshold for discriminating a positive-negative results was set at Cq 38.

### Determination of *Coxiella burnetii* as presumptive causative agent in the etiologic diagnosis of abortion

The World Organization for Animal Health (OIE) states that for abortions caused by *C. burnetii* the bacterial load is predictive of causality in individual animals. Indeed, a threshold of 10^4^ bacteria per gram of tissue has been proposed for discrimination of *C. burnetii* as the causative agent of abortion (16, 17). Based on experiments conducted in our laboratory, the threshold of 10^4^ *C. burnetii* per gram of tissue was set at a Cq value of 33, considering its role as a presumptive causative agent of abortion when Cq value was below 33. The definitive etiologic diagnosis could not be defined in this study, as it should be supported by the combination of detection of the abortifacient agent and demonstration of compatible lesions by histopathology.

### Statistical analysis and figures visualization

All data were included into a computerized spreadsheet (Microsoft Office Excel) and analyzed with R v4.1.3 (18).

Frequency analyses based on qPCR results were performed for the entire set of pathogens. The evaluated variables, i.e., small ruminant species (sheep-goat) and geographic area (Areas 1–5), were expressed as the percentage of positives and compared using the Fisher's exact test. For the assessment of the distribution of Cq values from each pathogen a histogram and a Shapiro-Wilk's test were performed. For those pathogens with a prevalence below 5% no statistical analysis was carried out due to lack of variability.

Of the 392 clinical cases referred to the laboratory during the evaluated period, a selection of 98 clinical cases was made to evaluate the usefulness of fetal tongues and placental swabs—alternative method—for the replacement of placental tissues and whole fetus—classical method—for the detection and quantification of the main pathogens causing abortions in small ruminants. For this optimization of the sampling procedure, we included a subset of 82 pairs of fetus-tongue and 72 pairs of placenta-placental swab.

The agreement between results of the sample pairs was performed on a dichotomous—positive/negative—scale. Inter-rater agreement analyses were performed for each pathogen using Cohen's Kappa (κ), including its 95% confidence interval (95% CI). Interpretation of Kappa values to assess the strength of agreement between techniques was based on the one proposed by Altman (19), which is as follows: κ ≤ 0.20 = poor, 0.21–0.40 = fair, 0.41–0.60 = moderate, 0.61–0.80 = good and 0.81–1.00 = very good. For *C. burnetii*, given the importance of the bacterial load in its clinical significance, we performed the agreement analysis for the whole set of cases positive to *C. burnetii* and split by its concentration into high (Cq ≤ 33) and low (Cq > 33).

Plots were produced using the ggplot2 package v3.3.3 (20), and further modified using the software Inkscape v1.2.1 (https://inkscape.org/). The level of statistical significance was represented with asterisks: four asterisks (****) indicated a *p*-value < 0.0001; three asterisks (***) indicated a *p*-value between 0.0001 and 0.001; two asterisks (**) indicated a *p*-value between 0.001 and 0.01; one asterisk (*) indicated a *p*-value between 0.01 and 0.05.

## Results

### Frequency of infectious agents detected in abortions in small domestic ruminants in the Iberian Peninsula

**Figure 2:**
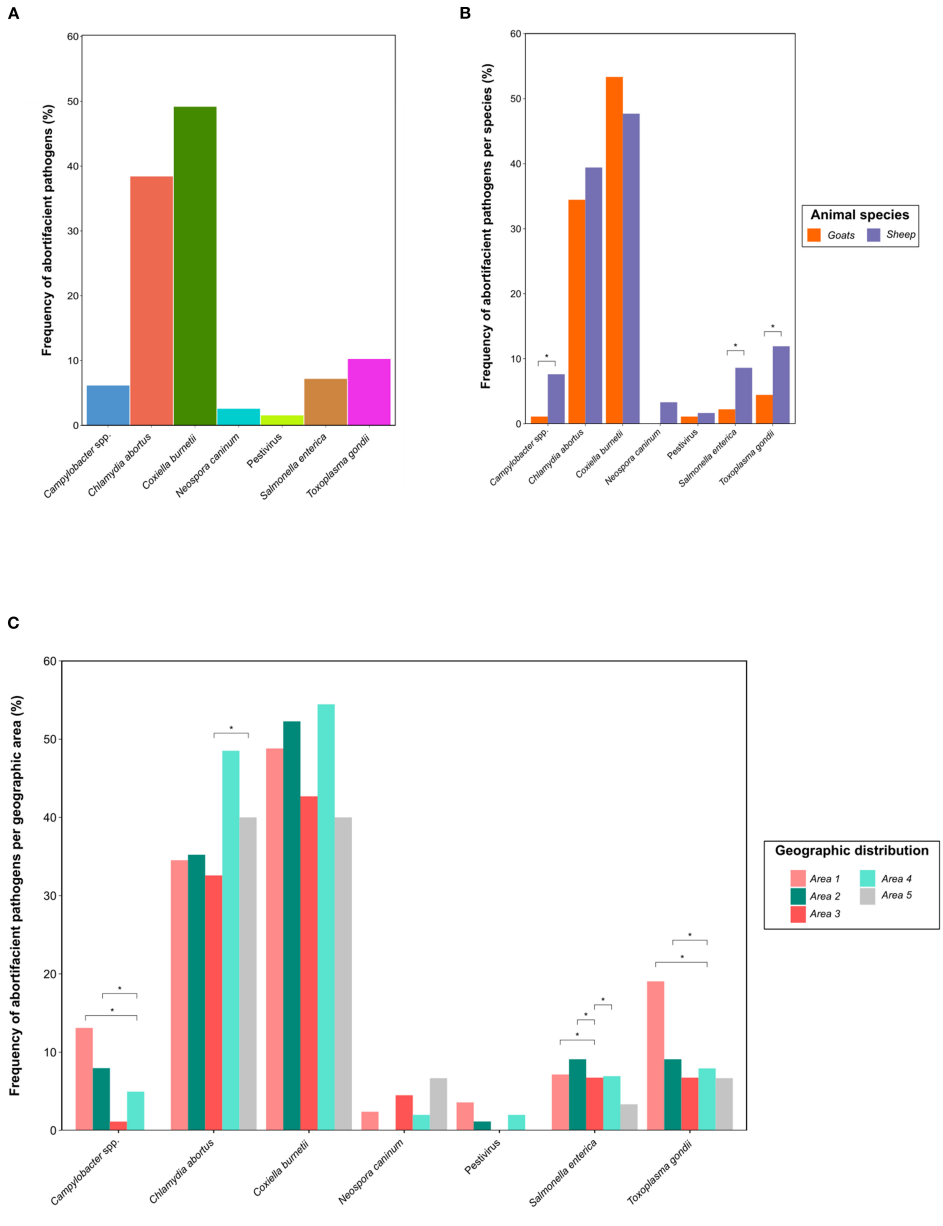
Distribution of 392 clinical cases of abortions in small domestic ruminants in the Iberian Peninsula referred to the diagnostics laboratory between April 2020 and May 2021 by **(A)** pathogen; and, within each pathogen by **(B)** small ruminant species, i.e., sheep (302) and goats (90); and **(C)** geographic area (Areas 1–5). The differences between sheep and goats and among geographic areas were evaluated by the Fisher's exact test. The level of statistical significance was represented with asterisks: two asterisks (**) indicated a *p*-value between 0.001 and 0.01; one asterisk (*) indicated a *p*-value between 0.01 and 0.05.

When evaluating the frequency of pathogens causing abortions in small ruminants during the evaluated period, *C. burnetii* and *C. abortus* were the most frequently detected pathogens in the 392 clinical cases referred to the laboratory, with a frequency of 49.0 and 38.3%, respectively (Figure 2A). To a lesser extent, *T. gondii* (10.2%), *S. enterica* (7.1%) and *Campylobacter* spp. (6.1%) were also detected in these clinical cases. *N. caninum* was involved in only ten cases (2.6%), while barely six cases were associated with Pestivirus infection (1.5%). None of the pathogens evaluated was detected in 24.0% of the clinical cases during this period.

Regarding to the detection of multiple microorganisms in the same clinical case, we appreciated that in 32.7% of abortion cases at least two different pathogens were detected, reducing this percentage to 6.1% for three agents. The most frequent combination of pathogens was the simultaneous detection of *C. burnetii* and *C. abortus*, reporting this finding in 20.9% of the cases, alone or in combination with other pathogens. Interestingly, the presence of *C. burnetii* and *C. abortus* alone accounted for 17.6 and 16.6% of all clinical cases, respectively. A summary of the 27 combinations of abortifacient pathogens detected in the clinical cases referred to the diagnostics laboratory during the evaluated period is available in Supplementary Table S1.

### Frequency of abortifacient pathogens in small domestic ruminants according to the ruminant species and the geographic area

Of the 392 clinical cases referred to the diagnostic laboratory, 302 cases were sheep abortions, while only 90 cases belonged to goats. We observed a significantly higher (*p* < 0.05) frequency of *T. gondii, S. enterica* and *Campylobacter* spp. in sheep abortions compared to goats. In contrast, we observed that the prevalence of *C. burnetii* was higher in goats (53.3%) than sheep (47.7%) abortions, but without significant differences. A detailed distribution of the differential frequency of abortifacient pathogens in sheep and goats and their statistical significance is available in Figure 2B.

According to the geographic area from which the clinical cases were referred (Figure 2C), we observed that cases from northern Spain (Areas 1 and 2) exhibited a significantly higher frequency of *Campylobacter* spp. and *T. gondii* (*p* < 0.05) than abortions from southeastern Spain (Area 4). The frequency of *C. abortus* was significantly higher (*p* < 0.05) in Portugal (Area 5) than in southwestern Spain (Area 3). For clinical cases positive to *S. enterica*, its presence was lower (*p* < 0.05) in southwestern Spain (Area 3) than in the other Spanish regions (Areas 1, 2, and 4). No significant differences were observed for *C. burnetii*.

### Differential distribution in the cycle of quantification for each abortifacient pathogen

**Figure 3:**
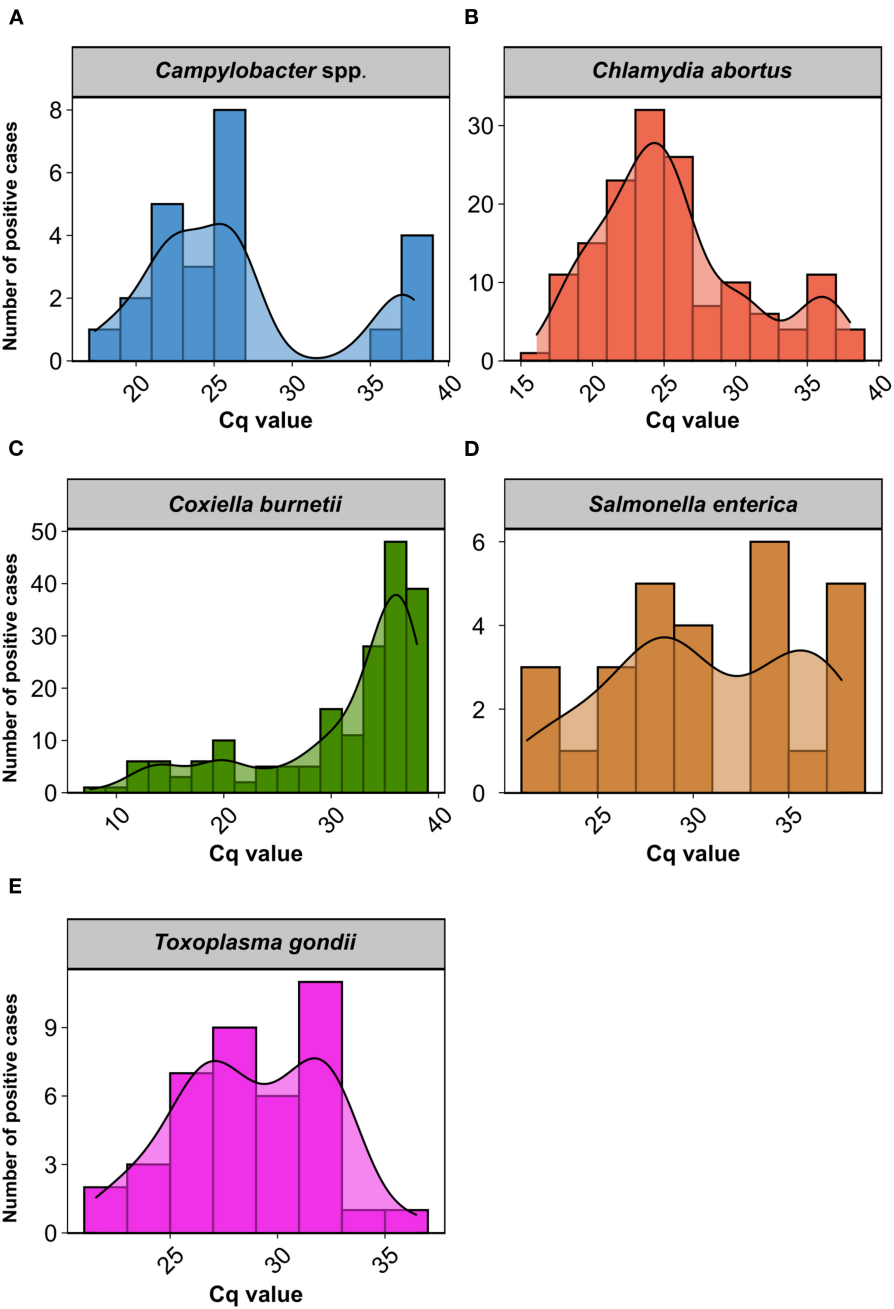
Histogram and density plot representing the distribution of cycle of quantification (Cq) values in clinical cases of abortion in small ruminants positive to **(A)** *Campylobacter* spp.; **(B)** *Chlamydia abortus*; **(C)** *Coxiella burnetii*; **(D)** *Salmonella enterica*; and **(E)** *Toxoplasma gondii*.

When evaluating the Cq distribution of each abortifacient pathogen in the clinical cases referred to the laboratory, we observed that the distributions varied depending on the microorganism evaluated (Figure 3). The most interesting finding was that, for *C. burnetii*, we observed a left-skewed histogram, with 60.4% of clinical cases in which its concentration was above the established threshold, i.e., Cq 33, for discriminating its presumptive role as causative agent of abortion.

A normal distribution for Cq values (*p* > 0.05) was only observed for *T. gondii*. In clinical cases positive for *C. abortus*, although we did not observe a normal distribution (*p* < 0.0001), in the histogram we could observe that most cases were normally distributed in a Cq range of 15–33, with a slight upturn of cases with Cq values higher than 33. Likewise, we appreciated a bimodal distribution for Cq values in *Campylobacter* spp. (*p* < 0.01) and *S. enterica* (*p* < 0.05), which was particularly evident in the former. *N. caninum* and Pestivirus were excluded from the comparison due to the reduced number of positive cases. A detailed information of Cq values for each pathogen and clinical case is available in Supplementary Table S2.

### Evaluation of the use of fetal tongues and placental swabs as an alternative sampling method for molecular diagnosis of abortion

Good to very good concordance in the Cohen's Kappa coefficient was observed for all pathogens when assessing the positive/negative concordance between the pathogens detected in the pairs of samples evaluated, i.e., fetus—tongue tissue and placenta—placenta swab, as reflected in Table 1, with the exception of *C. burnetii*. In the latter, although significant (*p* < 0.01), a fair concordance was revealed in the sample pair fetus—tongue, while it was moderate in the placenta—placenta swab pair.

**Table 1:** Two-way contingency table for the comparison of the classical (fetus and placenta) and the alternative method (fetal tongue tissue and placental swab) for etiologic diagnosis of abortions in small domestic ruminants.

		**Classical: positive**		**Classical: negative**					
**Pathogen**	**Comparison group (classical - alternative)**	**Alternative: positive**	**Alternative: negative**	**Alternative: positive**	**Alternative: negative**	**Cohen's kappa coefficient**	**95% CI**	***p*** **-value**	**Interpretation** ^a^
*Campylobacter* spp.	Fetus - fetal tongue	12	0	1	69	0.95	0.86–1	< 0.0001	Very good
	Placenta-placental swab	12	2	2	57	0.86	0.71–1	< 0.0001	Very good
*Chlamydia abortus*	Fetus - fetal tongue	20	1	1	60	0.94	0.85–1	< 0.0001	Very good
	Placenta-placental swab	20	1	2	49	0.90	0.79–1	< 0.0001	Very good
*Coxiella burnetii*	Fetus - fetal tongue	18	13	14	37	0.30	0.09–0.51	< 0.01	Fair
	Placenta-placental swab	24	14	7	27	0.42	0.22–0.63	< 0.001	Moderate
*Neospora caninum*	Fetus - fetal tongue	1	0	0	81	1.00	1	< 0.05	Very good
	Placenta-placental swab	3	0	0	69	1.00	1	< 0.0001	Very good
Pestivirus	Fetus - fetal tongue	6	0	0	76	1.00	1	< 0.0001	Very good
	Placenta-placental swab	2	0	0	70	1.00	1	< 0.0001	Very good
*Salmonella enterica*	Fetus - fetal tongue	10	1	0	71	0.95	0.84–1	< 0.0001	Very good
	Placenta-placental swab	8	2	1	61	0.82	0.62–1	< 0.0001	Very good
*Toxoplasma gondii*	Fetus - fetal tongue	17	2	1	62	0.90	0.78–1	< 0.0001	Very good
	Placenta-placental swab	18	0	0	54	1.00	1	< 0.0001	Very good

^a^Interpretation: poor (κ ≤ 0.20), fair (κ 0.21–0.40), moderate (κ 0.41–0.60), good (κ 0.61–0.80) or very good (κ 0.81–1.00).

Interestingly, the categorization of clinical cases positive for *C. burnetii* into high-low concentration based on Cq values (Table 2), evidenced that the discrepancies observed within sample pairs was determined by cases in which *C. burnetii* was present in low concentrations. Indeed, a very good concordance was observed for cases with Cq ≤ 33, while a no significant Cohen's Kappa coefficient was observed for clinical cases with Cq > 33.

**Table 2:** Two-way contingency table for the comparison of the classical (fetus and placenta) and the alternative method (fetal tongue tissue and placental swab) for etiologic diagnosis of *C. burnetii*.

		**Classical: positive**		**Classical: negative**					
**Pathogen**	**Comparison group (classical - alternative**	**Alternative: positive**	**Alternative: negative**	**Alternative: positive**	**Alternative: negative**	**Cohen's kappa coefficient**	**95% CI**	***p*** **-value**	**Interpretation** ^a^
High Cq value^b^	Fetus - fetal tongue	5	11	13	34	0.03	−0.21–0.28	0.75	Poor
	Placenta-placental swab	10	11	7	26	0.27	0.01–0.53	0.07	Moderate
Low-medium Cq value^c^	Fetus - fetal tongue	13	2	1	28	0.85	0.68–1	< 0.0001	Very good
	Placenta-placental swab	14	3	0	21	0.84	0.66–1	< 0.0001	Very good

^a^Interpretation: poor (κ ≤ 0.20), fair (κ 0.21–0.40), moderate (κ 0.41–0.60), good (κ 0.61–0.80) or very good (κ 0.81–1.00).

^b^High Cq value: Cq ≥ 33. Clinical cases with Cq < 33 were omitted from this two-way contingency table.

^c^Low-medium Cq value: Cq < 33. Clinical cases with Cq ≥ 33 were omitted from this two-way contingency table.

**Figure 4:**
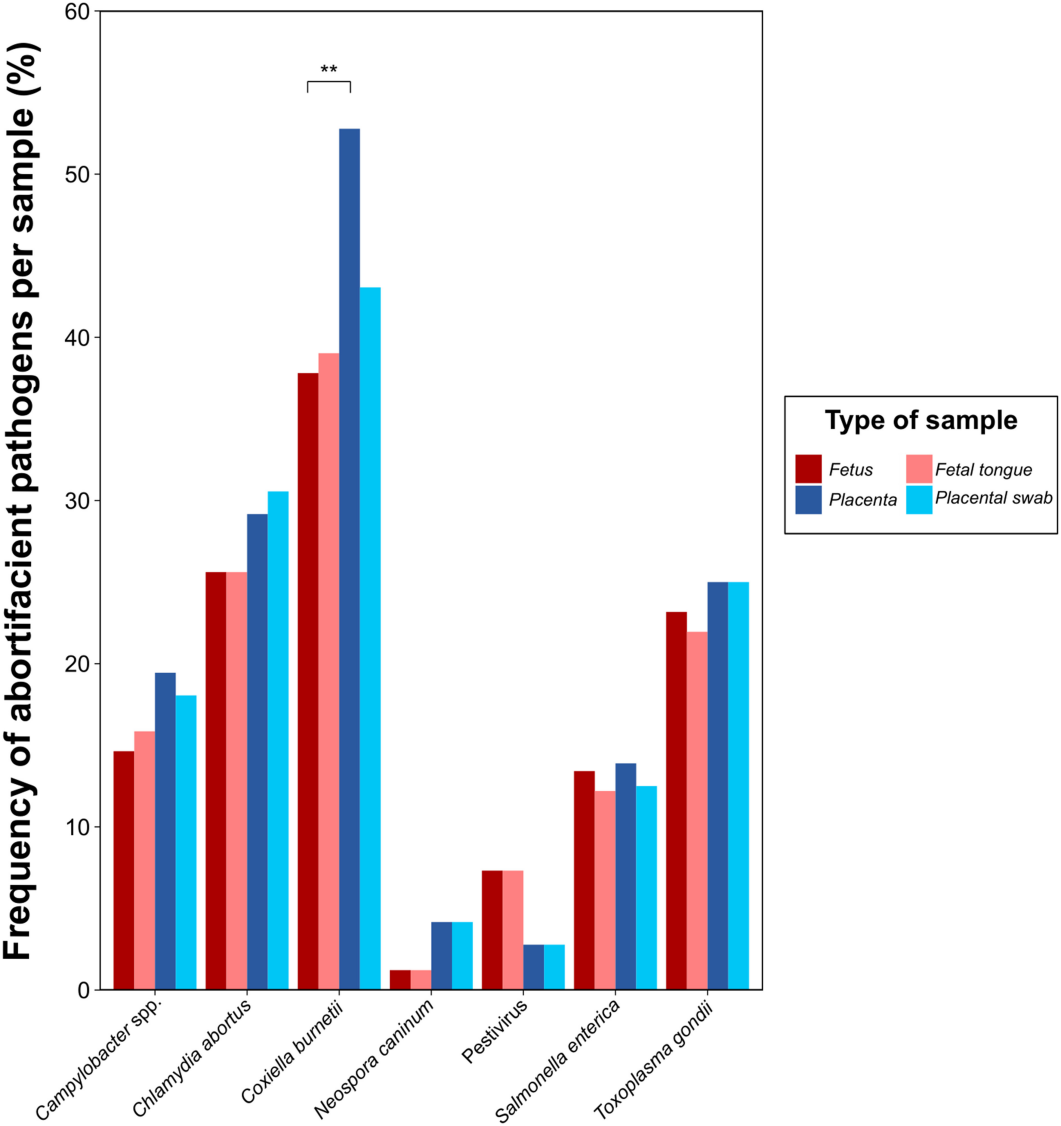
Frequency of abortifacient pathogens in small domestic ruminants in a selection of 98 clinical cases referred to the diagnostics laboratory from April 2020 to May 2021 for the comparison of the classical (fetus and placenta) and the alternative sampling method (fetal tongue tissue and placental swab) for molecular diagnosis. The level of statistical significance was represented with asterisks: two asterisks (**) indicated a *p*-value between 0.001 and 0.01; one asterisk (*) indicated a p-value between 0.01 and 0.05.

We further analyzed the differential distribution of the pathogens included in the study among the samples evaluated, i.e., pooled fetus, fetal tongue, placental tissue and placental swab, shown in Figure 4. Of note here is the significantly higher presence of *C. burnetii* in placental tissue compared to the fetus (*p* < 0.01), with no further differences for any other sample pair in any other microorganism evaluated.

## Discussion

Infectious abortions have an important impact on small domestic ruminant farms, i.e., sheep and goats, both in terms of profitability and health status (21, 22). This study evidenced that bacterial pathogens are mainly involved in abortions in small domestic ruminants in the Iberian Peninsula, with *C. burnetii* and *C. abortus* as the most frequent abortifacient agents. Here, we also demonstrated that the combination of fetal tongue tissue and placental swabs as an alternative sampling method for pathogen detection by qPCR presents results similar to those of whole fetus and placenta, revealing its usefulness as a rapid and sensitive sampling approach for the molecular diagnosis of abortions in small ruminants.

The selection performed in this study of the most frequent abortifacient pathogens associated with small domestic ruminants in Europe allowed us to detect pathogens involved in abortions in around 75% of cases, higher rates than most of previous case studies (23–25). In contrast, no agent could be detected in about a quarter of the cases, which may either be associated with non-infectious agents, such as toxics, nutritional disorders, genetic diseases, stress or extreme climatic conditions (26, 27) or, to a lesser extent, less frequent pathogens that may cause abortions secondarily (8, 13). The high detection rate of abortifacient pathogens may be explained by the high sensitivity provided by the quantitative molecular approach of this study, which can detect almost 100 copies of the pathogen per reaction. However, despite it is desirable to achieve this diagnostic depth, the detection of microorganisms at such low concentration should be carefully considered, since their role in abortion may not be clear, just as it has been previously proposed for *C. burnetii* (16, 17).

*C. burnetii, C. abortus* and, to a lesser extent, *T. gondii* were the most frequent abortifacient pathogens in the Iberian Peninsula in the evaluated period. Previous studies carried out in Spain (14, 28, 29) and Europe (13, 30) showed widely different prevalence rates among regions and countries for abortifacient pathogens, but all of them pointing out to these three agents as the most important microorganisms involved in small ruminant abortions in the continent. Prevalence differences among studies and absolute values should be considered carefully, given that different epidemiological contexts, study designs or diagnostic approaches are used (28). Indeed, most of the studies determine the seroprevalence of the abortifacient pathogens in the flock, but not its molecular identification in the fetus or the placenta, as performed in this study. This approach allowed us to provide a more accurate result, since serology only reveals prior exposure to the agent and does not necessarily imply that the pathogen detected in serology is the causative agent of the abortion.

A higher frequency of *T. gondii, S. enterica*, and *Campylobacter* spp. was observed in sheep abortions compared to goats, in contrast to previous studies, which point out a higher infection rate and susceptibility to abortions in goats (22, 31). Several studies have also described a higher susceptibility to *C. burnetii* in this species (4, 32) and, although non-significant, we appreciated a higher percentage of *C. burnetii* in goat abortions. The lack of concordance of our results with previous studies between small ruminant species may be explained by the limited number of goat abortions included in this study, which may be related to the reduced census of this species in Spain and Portugal when compared to sheep (33).

Regarding the geographic distribution of abortifacient pathogens in small domestic ruminants in the Iberian Peninsula, it is noteworthy the higher frequency of *Campylobacter* spp. and *T. gondii* in abortions in northern Spain. Several studies have pointed out that nutritional, climatic or management factors may influence the differential distribution of these pathogens among regions (12, 14, 32, 34). In this sense, for instance, a more frequent presence of *T. gondii* in this region may be determined by climatic conditions, since it has been described that a humid climate, as the one existing in the northern region of the Iberian Peninsula, is associated with greater oocyst survival in the environment (35, 36). Meanwhile, the distribution of *Campylobacter* spp. abortions in the Iberian Peninsula could be associated with a dissimilar frequency of abortive genetic clones among regions, as it has been observed between the United States and the United Kingdom for *C. jejuni* abortions in sheep (37). This genetic diversity could not be evaluated in the current study, as bacterial isolation was not performed. Therefore, further investigations need to be performed to evaluate factors that may be involved in the uneven distribution of abortion pathogens in the Iberian Peninsula.

This study evidences that co-infections in clinical cases of abortion are relatively frequent in small ruminants, as it has been demonstrated in recent studies (21, 38). However, based on the semiquantitative results provided by Cq values, we observed that in most cases a single microorganism was detected in a sufficiently high concentration to be considered as potential causative agent of the abortion by itself, unlike in digestive or respiratory processes, in which more than one pathogen are frequently involved (39–41). This finding may be due to the ubiquitous nature of some pathogens involved in abortions. Thus, for instance, for *S. enterica* and *Campylobacter* spp., although certain serotypes and species are considered abortifacient, such as *S*. Abortusovis or *C. fetus*, there are others which not only produce abortions, but can also be present in the intestinal microbiota in small ruminants, e.g., *S*. Typhymurium or *C. jejuni* (4, 42) and, consequently, be detected in the abortion in low concentrations as a consequence of a fecal contamination. Nonetheless, given the complexity of interactions among abortifacient pathogens and the uneven distribution of these agents in the fetus and placenta (8), which may lead to their underdetection, the identification of a pathogen as a causative agent of abortion should be carefully considered. Further research is needed to evaluate the role of low pathogen concentrations and co-infections in small ruminant abortions.

The sample selection made in this study for the development of an alternative sampling method, i.e., tongue tissue and placental swabs, for the detection of the main abortifacient pathogens in small domestic ruminants *via* qPCR, was determined by the differential tropism of these pathogens in the abortions, as demonstrated, for instance, for *C. burnetii*, with a higher tropism in placental samples (43). The replacement of pooled fetal organs by fetal tongues analysis was based on (i) the highly vascularized nature of this muscle (44), which allows the detection of any abortifacient pathogen that may have hematogenous dissemination and fetal tropism, and (ii) its intimate contact with the amniotic fluid, in which pathogens with placental tropism, such as *C. abortus*, are present (45). Meanwhile, the use of placental swabs recovered from the cotyledons and the inter-cotyledonary area of the placenta proved to be sufficiently representative to avoid manipulating the entire placenta. Indeed, good to very good agreement was demonstrated for the pairs of samples evaluated for each pathogen, except for *C. burnetii*.

*C. burnetii* is a very ubiquitous and highly resistant pathogen in the farm environment whose detection does not necessarily imply its causative role in abortion (14). In this study, we demonstrated that in more than half of the clinical cases in which *C. burnetii* was detected, its concentration was low enough to consider that its role in the abortion may not be determinant, in accordance with previous studies (21, 30). Interestingly, for this agent, a high concordance between the alternative and classical sampling methods was observed when its concentration was within the threshold to be considered as likely causative agent of abortion, but with not good agreement when its concentration was close to the detection limit of the technique. These facts demonstrate that the use of fetal tongue tissue and placental swabs in replacement of the whole fetus and placenta can be used as a rapid sampling approach for the molecular detection of the main pathogens involved in small ruminant abortions and, thus, minimizing biosafety risks for veterinarians, farmers and laboratory staff.

## Conclusions

This study reveals a high frequency of infectious abortions in small domestic ruminants in the Iberian Peninsula, with a particularly high presence of *C. abortus* and *C. burnetii* and an uneven distribution of pathogens between sheep and goats and among geographic areas. *C. burnetii* seems to have a controversial role in the etiology of the abortion, since in most of the cases its Cq value was upper the threshold set to be considered as a presumptive determinant of abortion. Furthermore, we demonstrate for the first time that the use of fetal tongue tissue and placental swabs as an alternative sampling method for the molecular diagnosis of abortions in small ruminants offers similar results to those provided by the use of the whole fetus and placenta. This new sampling approach will optimize diagnostic procedures, helping to provide rapid and sensitive results while minimizing biosafety risks.

## Data availability statement

The original contributions presented in the study are included in the article/Supplementary material, further inquiries can be directed to the corresponding author.

## Ethics statement

Ethical review and approval was not required for the study on animals in accordance with the local legislation and institutional requirements. Written informed consent from the owners for the participation of their animals in this study was not required in accordance with the national legislation and the institutional requirements.

## Author contributions

Study design was performed by GC and AB. Sample manipulation and laboratory analyses were performed by OA. Data analyses were performed by LD with contribution from OM-A. GC, AB, and OM-A provided technical and scientific support on the analysis. All authors participated in the manuscript writing or contributed to its revision and approving this final version.
