# Supplementary material for: Infectious abortions in small domestic ruminants in the Iberian Peninsula: Optimization of sampling procedures for molecular diagnostics
Source: Front Vet Sci. 2023 Mar 9;10:1152289. doi: 10.3389/fvets.2023.1152289 (PMC10033884; doi:10.3389/fvets.2023.1152289)
Supplement: Supplementary file 1 [file Table_1.DOCX]

Supplementary Material

**Infectious abortions in small domestic ruminants in the Iberian Peninsula: Optimization of sampling procedures for molecular diagnostics**

**Alzuguren, Oihane^1†^; Domínguez, Lara^1†^; Chacón, Gema^1^; Benito, Alfredo A.^1^; Mencía-Ares, Oscar^1*^**

^1^EXOPOL S.L., Pol. Río Gállego D/S, San Mateo de Gállego, 50840 Zaragoza, Spain**.**

^†^These authors contributed equally to this work and share first authorship

# * Correspondence:

# Oscar Mencía-Ares: [omencia@exopol.com](mailto:omencia@exopol.com)

**Supplementary Data**

**Table S1.** Combinations of abortifacient pathogens detected in 392 clinical cases referred to the diagnostics laboratory from April 2020 to May 2021.

| **Combination of abortifacient pathogens** | **n** | **Percentage (%)** |
| --- | --- | --- |
| *Coxiella burnetti* | 69 | 17.6 |
| *Chlamydia abortus* and *Coxiella burnetii* | 66 | 16.8 |
| *Chlamydia abortus* | 65 | 16.6 |
| *Toxoplasma gondii* | 17 | 4.3 |
| *Coxiella burnetii* and *Salmonella enterica* | 12 | 3.1 |
| *Coxiella burnetii* and *Toxoplasma gondii* | 12 | 3.1 |
| *Campylobacter* spp. and *Coxiella burnetii* | 7 | 1.8 |
| *Campylobacter* spp. | 7 | 1.8 |
| *Salmonella enterica* | 7 | 1.8 |
| *Campylobacter* spp., *Chlamydia abortus* and *Coxiella burnetii* | 4 | 1.0 |
| *Chlamydia abortus*, *Coxiella burnetii* and *Toxoplasma gondii* | 4 | 1.0 |
| *Neospora caninum* | 4 | 1.0 |
| *Campylobacter* spp., *Coxiella burnetii* and Pestivirus | 3 | 0.8 |
| *Campylobacter* spp., *Coxiella burnetii* and *Toxoplasma gondii* | 3 | 0.8 |
| *Chlamydia abortus*, *Coxiella burnetii* and *Neospora caninum* | 3 | 0.8 |
| *Campylobacter* spp., *Coxiella burnetii* and *Salmonella enterica* | 2 | 0.01 |
| *Chlamydia abortus*, *Coxiella burnetii* and *Salmonella enterica* | 2 | 0.01 |
| *Coxiella burnetii*, *Salmonella enterica* and *Toxoplasma gondii* | 2 | 0.01 |
| *Campylobacter* spp. and *Chlamydia abortus* | 1 | 0.003 |
| *Chlamydia abortus* and *Salmonella enterica* | 1 | 0.003 |
| *Chlamydia abortus* and *Toxoplasma gondii* | 1 | 0.003 |
| *Neospora caninum* and *Salmonella enterica* | 1 | 0.003 |
| Pestivirus | 1 | 0.003 |
| Pestivirus and *Salmonella enterica* | 1 | 0.003 |
| *Coxiella burnetii* and *Neospora caninum* | 1 | 0.003 |
| *Coxiella burnetii, Neospora caninum* and *Toxoplasma gondii* | 1 | 0.003 |
| *Coxiella burnetii* and Pestivirus | 1 | 0.003 |

**Table S2.** Detailed information of 392 clinical cases referred to the diagnostics laboratory from April 2020 to May 2021, including the pathogens detected and the value of the cycle of quantification (Cq). Format .xlsx
